# Hydrops fetalis with isolated massive ascites in a preterm neonate with rhesus disease

**DOI:** 10.1007/s10354-021-00829-7

**Published:** 2021-03-18

**Authors:** Nasenien Nourkami-Tutdibi, Martina Geipel, Gabriele Meyberg-Solomayer, Zoltan Takacs, Sascha Meyer

**Affiliations:** 1 Hospital for General Pediatrics and Neonatology, Saarland University Medical Center, 66421 Homburg/Saar, Germany; 2 Hospital of Gynecology, Obstetrics and Reproductive Medicine, Saarland University Medical Center, Homburg/Saar, Germany

**Keywords:** Erythroblastosis fetalis, Hemolytic disease of newborn, Anemia, Neonatal intensive care units, Jaundice, neonatal

## Abstract

Significant progress in prenatal care has decreased the incidence of rhesus incompatibility, which may result in hemolytic disease of the fetus and newborn (HDFN). This case report describes an unusual presentation of HDFN in a preterm infant delivered by caesarean section with isolated massive abdominal fluid collection as the leading clinical sign in addition to severe anemia. The immediate drainage of ascites provided transient clinical stabilization with improved pulmonary function in the delivery suite. After admission to the neonatal intensive care unit (NICU), HDFN treatment was initiated. This case report shows the importance of adequately trained staff including neonatologists, pediatricians and NICU nurses in the delivery suite to provide neonatal intensive care for HDFN.

Rhesus incompatibility in pregnancy may result in hemolytic disease of the fetus and newborn (HDFN) [1]. Significant progress in prenatal care strategies and improved prenatal management options have led to a remarkable reduction in perinatal mortality [2, 3]. HDFN can present with a variety of clinical features [4].

Here, we present a preterm neonate with HDFN born at a gestational age of 31 + 4 weeks by caesarean section because of hydrops fetalis and non-reassuring cardiotocography (CTG) with a weight of 2200 g to a GII, PI mother. Due to severe anemia secondary to rhesus disease, packed red blood cells were transfused in utero three times. Immediately after birth, the infant demonstrated signs of severe respiratory compromise requiring intubation (at 3 min of life) following administration of surfactant. On physical exam, gross enlargement of the abdominal cavity was noted with massive fluid collection (Fig. 1a) and hepatomegaly, mandating the immediate drainage (at 10 min of life) of 200 mL of ascites (Fig. 1b). In parallel, the neonate received fluid resuscitation and packed red blood cell transfusion in the delivery suite due to severe anemia with an initial hemoglobin concentration of 5.4 g/dl.

**Fig. 1:**
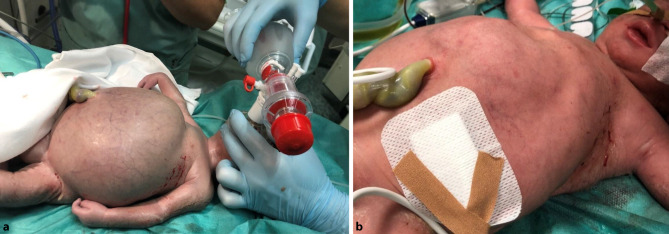
**a** Severe anemia and massive abdominal distension due to ascites immediately after birth. **b** Normalization of skin color following intubation, transfusion of packed red blood cells and reduction in abdominal circumference after drainage of ascites in the delivery room

After admission to our neonatal intensive care unit (NICU), the neonate required inhaled nitric oxide for persistent pulmonary hypertension, phototherapy and administration of immunoglobulins (total dose of 2 g/kg body weight) for hemolytic rhesus disease (positive Coombs test; admission serum LDH concentration of 10,000 U/L; maximum serum bilirubin concentration of 210.8 µmol/L within the first 12 h of life). As serum bilirubin was below the level for exchange transfusion after the initial phase, phototherapy was continued, and no exchange transfusion was necessary.

On ultrasonography and echocardiography, no pleural or pericardial effusions were initially noted. Despite hypertrophic cardiomyopathy, left ventricular function was initially good and no soft tissue edema was seen postnatally (Fig. 1). Transfusion of platelets, red blood cells and fresh frozen plasma were required within the first days of life. During the further clinical course, cardiac function deteriorated secondary to worsened hypertrophic cardiomyopathy. On day 6 of life, the patient presented with sudden bradycardia and cardiac arrest. A pericardial effusion was successfully drained, but resuscitation was nevertheless unsuccessful. Autopsy revealed septic toxic cardiovascular failure due to necrotizing enterocolitis with extensive ischemic hemorrhagic of the intestinal mucosa.

This case shows an unusual presentation of HDFN in a preterm infant with isolated massive abdominal fluid collection as the leading clinical sign in addition to severe anemia in the delivery suite. The immediate drainage of ascites provided transient clinical stabilization with improved pulmonary function in the delivery suite. To provide adequate neonatal intensive care in the delivery suite in patients with HDFN, adequate staffing is of utmost importance; our patient was cared for by two fully trained neonatologists, one pediatrician, and one nurse with full training in neonatology. Because of the unusual presentation in our patient with possible further underlying disease (e.g., genetic, metabolic), genetic studies were performed, including exome analysis, which did not reveal any pathological findings.
